# Autologous fat grafting: Harvesting techniques

**DOI:** 10.1016/j.amsu.2018.11.005

**Published:** 2018-11-13

**Authors:** Tomás Fontes, Inês Brandão, Rita Negrão, Maria João Martins, Rosário Monteiro

**Affiliations:** a Departamento de Biomedicina - Unidade de Bioquímica, Faculdade de Medicina, Universidade do Porto, Porto, Portugal; b Instituto de Investigação e Inovação em Saúde (I3S), Universidade do Porto, Porto, Portugal; c Unidade de Saúde Familiar Pedras Rubras, Agrupamento de Centros de Saúde Maia-Valongo, Maia, Portugal

**Keywords:** Adipose tissue, Autologous fat graft, Reconstructive surgery, Plastic surgery, Harvesting techniques

## Abstract

Autologous fat grafting is widely used for soft-tissue augmentation and replacement in reconstructive and aesthetic surgery providing a biocompatible, natural and inexpensive method. Multiple approaches have been developed in the past years, varying in the location of adipose tissue donor-sites, use of wetting solutions, harvesting, processing and placing techniques. Despite many advances in this subject, the lack of standardization in the protocols and the unpredictability of the resorption of the grafted tissue pose a significant limitation for graft retention and subsequent filling. In this review, we discuss several approaches and methods described over the last years concerning the harvesting of autologous fat grafts. We focus on contents such as the best donor-site, differences between existing harvesting techniques (namely tissue resection, hand aspiration or liposuction techniques), recommended harvesting cannula diameters, pressure application and volume of wetting solution injected prior aspiration. Results and comparisons between methods tend to vary according to the outcome measured, thus posing a limitation to pinpoint the most efficient methods to apply in fat grafting. Additionally, the lack of a standard assay to determine viability or volume augmentation of fat grafting remains another limitation to obtain universally accepted grafting procedures and protocols.

## Highlights

• Distinct harvesting procedures associate with different outcomes of fat graft take.
• Flank, abdomen, thigh and knee are the more consistently used donor-sites for fat.
• Higher vacuum pressures in liposuction are more traumatic for the tissue.
• The tumescent technique is a safer procedure with improved aesthetic results.
• Comparing harvesting techniques is a big challenge given the multiple variables.

## 1 Introduction

The first record of an autologous fat transfer procedure dates back to 1893, when Neuber transferred autologous adipose tissue to a facial scar depression and documented the outcomes [1]. However, autologous fat grafting only came into play as an important and easily accessible filler with the advent of liposuction in the 1980s [2]. Since then, the number of applications reporting on its use has been growing, mostly because adipose tissue is readily available, natural, easy to harvest and inexpensive. In addition, being autologous, adipose tissue does not trigger an immunological response that could lead to rejection, besides associating with very little donor-site morbidity [3]. Furthermore, this tissue can be used not only as a volume replacer but also as a tissue quality improver [4]. Some clinical applications of autologous fat grafts comprise: a) aesthetic breast procedures (primary breast augmentation, replacement of previous breast implants, reversal of radiation damage after breast cancer treatment, breast reconstruction or recontouring after mastectomy and correction of deformities such as tuberous breast or brassiere strap grooves [[5], [6], [7], [8], [9]]; b) mastoidectomy, rhinoplasty, facial rejuvenation, hand rejuvenation, gluteoplasty and laryngoplasty [4,[10], [11], [12], [13], [14], [15], [16]]; c) improvement and/or correction of deformities observed in Parry-Romberg syndrome, Poland syndrome, Dupuytren's and Raynaud's diseases and *pectus excavatum* [[17], [18], [19], [20], [21], [22]]; d) treatment of scars, ulcers and burns [23,24].

Although it has been widely used for decades now, one of the main issues in autologous fat grafting is the unpredictable resorption after transplantation, which may require repeated injections and lead to poor results [25,26]. Therefore, the need of optimizing fat grafting methodology should be of maximal importance to minimize fat graft loss.

**Fig. 1:**
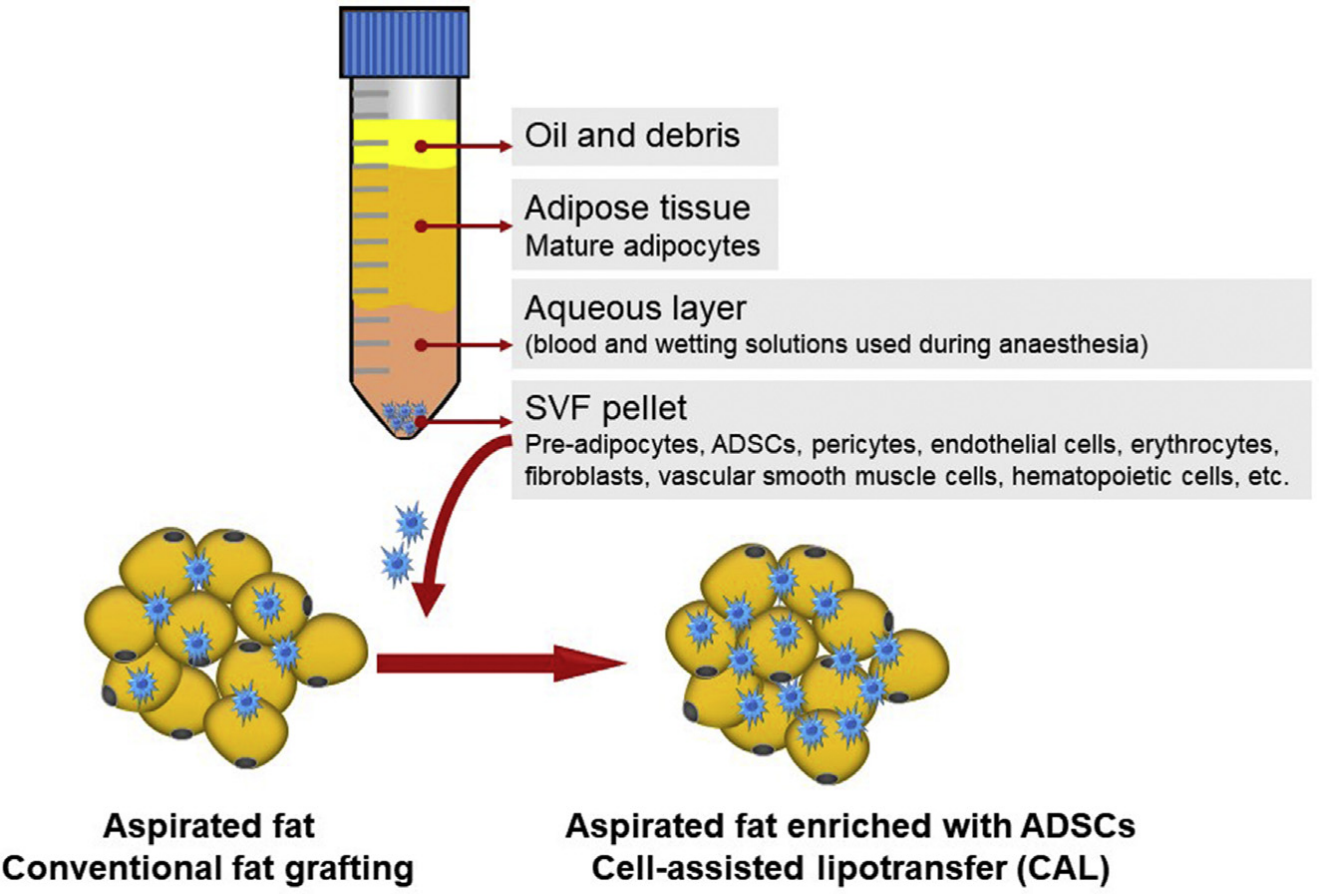
Lipoaspirate components and enrichment of the aspirated fat by cell-assisted lipotransfer (CAL). SVF, stromal vascular fraction; ADSCs, adipose derived stem cells.

Harvested adipose tissue is composed of mature adipocytes, extracellular matrix and a stromal vascular fraction (SVF), constituted by different cells including adipose derived stem cells (ADSCs), pericytes, endothelial cells, erythrocytes, fibroblasts, vascular smooth muscle cells, hematopoietic cells and other immune cells (Fig. 1) [2,27,28]. Noteworthy, recent reports have identified adipose tissue as the tissue in the body that contains the highest percentage of adult stem cells [29,30]. These ADSCs can undergo multilineage differentiation [[30], [31], [32], [33], [34], [35], [36], [37], [38]] and may be crucial for fat graft take since mature adipocytes that survive harvesting procedures will not replicate and will eventually die, generating harmful inflammatory responses [39]. Indeed, ADSC-enriched grafts observed in cell-assisted lipotransfer (CAL) have been associated with better graft viability and outcome after transplantation (Fig. 1) [28,40]. Yet, this is still a matter of debate, with other clinical studies claiming that there is no significant difference in the survival rate of the transplanted fat between conventional fat grafting and SVF-enriched fat grafting and even adding that postoperative complications are more often observed in the latter [41]. According to a recent meta-analysis by Laloze et al., that evaluated the efficacy of CAL by comparing 16 studies, the fat survival rate was significantly higher with CAL when compared with conventional procedures, independent of injection site (breast or face) but only for small injection volumes (below 100 mL). The same analysis concluded that CAL associates with more complications and did not decrease the number of additional surgical procedures needed after the first fat grafting [41].

So far, many techniques and procedures have been documented regarding the use of adipose tissue grafts in reconstructive surgery.

Distinct harvesting procedures result in different outcomes of fat graft take, as observed by multiple in vitro analyses, in vivo animal experiments and human studies [25]. Several variables need to be taken into account in order to get the highest cell viability and survival rates possible. Those include the body location for adipose tissue donor-site, the harvesting procedure, the harvesting cannula, the pressure applied or the possibility of injecting a tumescent solution with anesthetic before tissue collection. Herein, we will summarize the various reported approaches for harvesting during autologous fat grafting.

## 2 Harvesting

Distinct harvesting procedures lead to different outcomes of fat graft take. As aforementioned, details such as the best donor-site, what adipose tissue harvesting technique to use, what harvesting cannula diameter to choose, what pressure to apply to avoid the decrease of cell viability or the possibility of injecting a tumescent solution with anesthetics before tissue collection are taken into account in order to get the highest cell viability and survival rates possible.

### 2.1 What is the best donor site?

When it comes to choose the body location for harvesting, flank, abdomen, thigh and knee are the more consistently used donor-sites. Li et al. compared fat tissue grafts harvested from 6 women and different donor-sites (flank, upper and lower abdomen, lateral and inner thigh). The adipose tissue was implanted subcutaneously into nude mice and grafts were harvested and analyzed at 12 weeks. Authors found no significant differences among grafts from distinct donor sites regarding weight, volume and histological features (including integrity, cysts, inflammation, fibrosis and neovascularization). Also, the levels of cell surface markers and SVF did not differ. Thus, authors suggested that factors like accessibility and patient preference should be considered primordial criteria for donor-site selection [42]. Similarly, Ullmann et al. reported no difference in terms of weight, volume and histological features, such as vascularization and fibrosis, between fat from 3 donor sites (thigh, abdomen, and breast) from a 48 year-old woman that were implanted into a nude mice model [43]. Likewise, Lim et al. concluded that both abdominal and non-abdominal sources of fat presented equal success in correcting craniofacial soft-tissue deficiencies in 27 patients with craniofacial macrosomia (n = 19) or Treacher Collins syndrome (n = 8) [44]. This was also supported by Small et al. that found no difference in volume retention between fat harvested from abdomen or thighs, in a retrospective study involving 73 patients that underwent breast reconstruction [45].

On the other hand, Padoin et al. conducted a prospective cross-sectional study in 25 women that underwent liposuction in 4 or more distinct areas. Mesenchymal stem cell were extracted and quantified from lipoaspirates and results revealed a higher concentration of cells in samples collected from the lower abdomen and inner thigh when compared to those collected from the upper abdomen, trochanteric region, knee and flank [46].

Recently, Tsekouras et al., in a study involving 40 donor women, reported the outer thigh adipose tissue to have significantly higher SVF cell count in comparison to any other sites such as inner thigh, abdomen, waist and inner knee. Also, inner and outer thigh were associated with a significantly higher number of ADSCs when compared to abdominal, waist, and inner knee lipoaspirates [47].

Of note, Di Taranto et al. evaluated superficial and deep adipose tissue (SAT and DAT, respectively) collected from 16 female donors undergoing first-time liposuction. Additionally, full-thickness skin specimens from the abdominal wall were collected from 3 cadaver donors for histological and immunohistochemistry analysis of the hypodermal layers. Results revealed that SAT was homogeneously present in all body areas, whereas DAT was more abundant in the abdomen, hips, knee, peritrochanteric area, upper inner thigh, and posterior compartment of the arm. SVF cell fraction from abdominal SAT lipoaspirates showed higher viability and higher expression of both stem/stromal surface antigen endoglin (CD105) and vascular endothelial growth factor (VEGF) when compared with DAT from the same harvesting site. Overall, SAT was associated with better stem properties thus suggesting its preferable use as a donor site [48].

### 2.2 Harvesting procedures and pressure: impact on graft survival

After choosing the body donor-site, the next step is to choose the harvesting method. Direct excision, syringe hand aspiration and suction-assisted liposuction using various pressures (and also using different mechanisms to create that pressure) are some of the currently used techniques [[49], [50], [51]]. In addition, liposuction can also be assisted by a liquid-jet, ultrasound pulses or laser energy [[52], [53], [54]].

In order to perform liposuction or manual aspiration, negative pressures are required. Despite the divergence between harvesting techniques (and distinct pressures) suggested by different authors to obtain better functional grafts, it is well accepted that high vacuum pressures of conventional liposuction are more traumatic for the tissue leading to adipocyte structural disruption [55,56].

The different types of harvesting techniques with distinct pressures are summarized and compared in Table 1.

**Table 1:** Comparison between harvesting techniques and different pressures.

Techniques	Methods	Results	Reference
**Manual aspiration (syringe) and PAL (350 and 700 mmHg)**	Trochanteric fat harvested from 3 healthy patients aged 36, 43 and 58 years. Number of isolated SVF cells was assessed.	Cell yield with a pressure of 350 mmHg, assisted or not, was higher than that obtained at 700 mmHg. Cell yield with PAL (350 mmHg) was significantly superior to aspiration with a syringe (p < 0.05).	Mojallal et al. [57] Year: 2008
**Manual aspiration (275, 394 and 550 mmHg) and PAL (350 and 700 mmHg)**	Comparative study in 15 healthy man and women aged 25–60 years, undergoing abdominal cosmetic surgery. Samples underwent histological analysis in order to verify the integrity and functionality of the harvested adipocytes and ADSCs.	Values of negative pressure produced by the syringes as well as pressures of 350 and 700 mmHg obtained by PAL did not lead to differences in the number of adipocytes and viability of the ADSCs extracted.	Charles-de-Sá et al. [58] Year: 2015
**Manual aspiration and PAL (375 mmHg)**	Fat tissue was obtained from 9 donors undergoing abdominoplasty. Samples were divided into 2 fat sections, harvested using either manual aspiration or PAL. Number of isolated ADSCs was counted and proliferation rate and cell viability were assessed. The ability of isolated ADSCs to differentiate into mature adipocytes was analyzed by gene marker expression.	PAL revealed at least similar quality and quantity of ADSCs as manual aspiration. Cells harvested by PAL had higher expression levels of differentiation markers (e.g. adiponectin).	Keck et al. [51] Year: 2014
**PAL at high pressure (760 mmHg) and low pressure (250 mmHg)**	Abdominal lipoaspiration was performed on 3 patients on the opposite sides of the flank after infiltration with tumescent solution. Adipocyte survival and cell viability were measured in vitro.	Adipocyte count was 47% higher when aspirated at low pressure compared with high pressure, immediately after harvesting. Cell viability was significantly higher at day 7 with low-pressure aspiration.	Cheriyan et al. [59] Year: 2014
**PAL (** < **760 mmHg) and tissue resection**	6 healthy women underwent abdominoplasty surgery. Subcutaneous adipose tissue of the abdomen was analyzed. SVF isolated from abdominal fat harvested from patients (n = 6). Cell yield and viability of ADSCs were assessed. Cell phenotypes were determined by immunostaining and FACS, and doubling times were calculated. Senescence ratios of the cells were measured. Multipotency was evaluated by induced differentiation analyses.	No differences in multiplication rates, senescence ratios and multipotency of cultured ADSCs.	Barzelay et al. [50] Year: 2015
**PAL (760 mmHg) and tissue resection**	Adipose tissue obtained from paired tissue resection and PAL adipose tissue from the abdomen of 3 healthy women aged 26–54 years. In vitro analysis: samples were processed to isolate the SVF. ADSC yield and cell viability were assessed. Adipogenic and osteogenic differentiation capacity were assessed in vitro using phenotypic staining and quantification of gene expression. In vivo analysis: ADSCs were applied in an in vivo mouse model of tissue repair to evaluate their regenerative potential.	Lower ADSCs yield in SVF cells using PAL (42.4%) in comparison to tissue resection (55.8%). No difference in the other parameters.	Duscher et al. [60] Year: 2016
**WAL (375 mmHg) and manual aspiration (290 mmHg)**	8 women were included in the study and the two techniques were used for each patient. The lipoaspirates of subcutaneous abdominal fat were collected on both side of the umbilic in each patient. In vitro analysis: cell yield, viability and immunophenotype of the SVF fraction. Osteogenic and adipogenic differentiation and immunosuppressive capacity of ADSCs was assessed in vitro. In vivo analysis: immunosuppressive capacity of ADSCs during a delayed-type hypersensitive response model in mice.	Equivalent number of viable cells, fibroblast colony-forming units and immunophenotype. Interestingly, ADSCs isolated from manual liposuctions showed significantly higher immunosuppressive potential than those from WAL in vitro but not in vivo.	Bony et al. [61] Year: 2015
**LAL and PAL**	Fat tissue obtained from the breast of 7 men aged 19–24 years diagnosed with gynecomastia. In vitro analysis: cell yield, viability, pluripotency, surface markers expression and apoptosis of ADSCs were assessed.	No difference in surface and cellular differentiation markers. Lower number of viable ADSCs and higher apoptosis indicators in LAL, 24 h after harvesting, but these differences were reversed after 72 h.	Yildiz et al. [62] Year: 2016
	Fat tissue obtained from 12 healthy women between the ages of 33–55 years who were undergoing elective lipoaspiration of the abdomen. Each patient undergoing laser-assisted liposuction (n = 6) was matched for age (within 2 years) with a patient undergoing suction-assisted liposuction (n = 6). Age-matched patients underwent liposuction procedures on the same day. LAL and PAL could not be harvested from the same anatomical location. In vitro analysis: Cell yield, cell viability and proliferation, surface marker phenotype, osteogenic differentiation and adipogenic differentiation capacity of ADSCs. In vivo analysis: regenerative capacity of ADSCs in a cranial defect in nude mice.	All in vitro parameters such as cell yield, viability, proliferation and frequency of ADSCs were all significantly less with LAL compared to PAL. In vivo, ADSCs from LAL led to significantly less osseous healing in comparison to PAL.	Chung et al. [53] Year: 2013
**UAL and PAL**	Fat tissue obtained from 3 healthy women aged 28–48 years, undergoing elective liposuction of the abdomen. Two lipoaspirate samples were harvested from identical sites in each patient, with PAL being performed before UAL. In vitro analysis: Cell yield, viability and proliferation, surface marker phenotype, osteogenic, adipogenic and chondrogenic differentiation capacity of ADSCs. In vivo analysis: regenerative capacity of ADSCs in an excisional wound in nude mice	Equivalent results between both techniques. Cells harvested are suitable for cell therapy and tissue engineering.	Duscher et al. [63] Year: 2016

ADSCs, adipose derived stem cells; FACS, fluorescence-activated cell sorting; LAL, laser-assisted liposuction; PAL, power-assisted liposuction; SVF, serum vascular fraction; UAL, ultrasound-assisted liposuction; WAL, water-jet assisted liposuction.

### 2.3 What is the ideal cannula?

The characteristics of the cannula used to collect fat, mainly its diameter and number of holes, influence the success of fat graft procedures. Campbell et al. reported an inverted relationship between cellular damage and the diameter of the instrument used to extract fat [64].

Multi-perforated cannulas help reduce pressure on each hole, decreasing damage in the samples collected [65]. Trivisonno et al. compared 2 mm and 3 mm diameter cannulas, both with 170 mm length and a rounded tip. The 2 mm cannula had 5 round spirally placed ports, each with a 1 mm diameter, and the 3 mm cannula had a single side located 3 × 9 mm port. The 2 mm cannula concurrently facilitated harvesting from more superficial and vascularized layers of adipose tissue, and reduced patient discomfort and trauma. In addition, this 2 mm cannula was able to isolate more ADSCs, and with a higher potential for capillary-like structure formation than the 3 mm cannula. Nevertheless, ADSC viability, morphology and proliferation capacity did not vary significantly between the two cannulas [66]. Alharbi et al. compared a micro-harvesting 2 mm cannula with four 600 μm gauged orifices and a blunt tip with a conventional 3 mm single hole blunt tip cannula, with the first claiming significantly higher viability and migration of isolated ADSCs [67]. However, Rubino et al. concluded that fat harvested with a 3 mm cannula showed more adipocyte density than fat harvested with a 2 mm cannula [68]. Erdim et al. showed an increase in graft viability in fat harvested from 10 female patients using a 6 mm cannula during liposuction compared with grafts obtained by 4 mm and 2 mm cannulas [69]. A prospective study by Ozsoy et al. concluded that a greater number of viable adipocytes was obtained with a 4 mm-diameter cannula when compared with 2 or 3 mm cannulas [70].

Although the optimal cannula size still lacks consensus, it is well accepted that it should be large enough to avoid shear stress and to preserve adipocytes and SVF cells [[70], [71], [72]].

### 2.4 Is there a difference in wet or dry aspiration outcomes?

There are a few types of liposuction techniques according to the volume of injected solution into the fat donor-site. The dry technique consists of direct aspiration without injecting any preparation solution and it is nowadays obsolete due to blood loss that can account for 20–50% of the aspirated volume [25,[73], [74], [75]]. In the wet technique, proposed by surgeons Clayton and Hetter, the fat donor-site is injected with a wetting solution (which may contain saline, anesthetics and other substances) prior to aspiration, following a ratio of infiltrate volume: aspirated volume lower than 1:1, resulting in a blood loss of 4–30% of the aspirated volume [[75], [76], [77]]. Later, in the superwet technique proposed by Fodor et al., a ratio of infiltrate to total aspirate of 1:1 was used and was associated with reduced blood loss of 1–2% of the aspirated volume [43,75,76].

Finally, the tumescent technique, introduced by Klein, presented a large volume of infiltrate with a ratio of infiltrate volume to total aspirate volume of 2–3:1. This technique is accompanied by a reduced blood loss of around 1% of the aspirated volume and does not require general anesthesia, therefore being considered as the safer procedure for larger aspirations and with improved aesthetic results. Tumescent anesthesia must be injected 45 min before harvesting to ensure hydrodissection and bloodless collection [74,75,78].

Lidocaine alone has been associated with decreased adipocyte function, with Moore et al. finding transient changes to lipolysis and glucose transport in the presence of local anesthetic. Interestingly, removal of lidocaine through washing harvested lipoaspirate returned these levels to normal [79]. Tumescent anesthesia can also be a vehicle to drive substances with a given effect to the adipose tissue to be harvested (Table 2). Local anesthetics, either with or without vasoconstrictor agents were found not to have a significant effect on long-term survival of grafted fat, contradicting the hypothesis proposed by Moore et al. suggesting that lidocaine inhibited growth of adipocytes [79,80]. Agostini et al. verified that histomorphometric characteristics (like cross-sectional profile, cytoplasmic rim, connective tissue, amorphous ground substance, vacuoles, cytoplasmic swelling/disruption, apoptosis or necrosis) and cell viability did not significantly differ between dry and wet harvesting liposuction [73].

**Table 2:** Wetting solutions used in tumescent anesthesia.

Wetting solutions	Description	Examples
**Crystalloid solutions**	Diluents for anaesthetics.	**Isotonic saline 0.9% NaCl** (Neutralizes the acidic pH of lidocaine. Associated with burning sensation.) **Lactated Ringer's solution** (Does not cause burning sensation and reduces the sodium load.) [81,82]
**Vasoconstrictors**	Constrict the blood vessels, reduce blood loss, and ameliorate tissue perfusion with the anaesthetics by increasing the duration and the quality of anesthesia.	**Adrenaline (epinephrine)****l****-ornithine 8-vasopressin** [[81], [82], [83], [84]]
**Local anaesthetics**	Allow absence of pain sensation during liposuction.	**Lidocaine** **Prilocaine** **Articaine** [[85], [86], [87], [88]]
**Buffers**	Raise the pH of the solution and avoid the burning sensation. Augment the proportion of nonionized lipid soluble lidocaine, which can more rapidly enter the nerve cells.	**Sodium bicarbonate** [82,89]
**Antioxidants**	Reduce oxidative stress in the fat graft. Ameliorate survival of ADSCs and preserve graft volume.	**N-acetylcysteine** [90]

## 3 Conclusion

Autologous fat grafting has become increasingly used as a method for multiple volume filling applications. The major obstacle to the widespread of its clinical use is the lack of standardized guidelines during harvesting, processing and implantation steps [91]. Indeed, many authors have recognized that there is no universally accepted methodology for fat grafting [92,93].

Fat is a delicate tissue and must be handled with maximal care to maintain its viability [6]. The ideal methodology to approach autologous fat grafting has been a major focus in the last years, but patient-related factors should also be taken into account when designing a study.

Donor-site morbidity, like hematoma or, more frequently, local deformities caused by liposuction, and recipient-site complications, such as infections and, although very unlikely, pulmonary embolism, cardiac arrest, or deep venous thrombosis, represent drawbacks of adipose tissue transplant. Nevertheless, autologous fat grafting is reported to be a very safe procedure with very low morbidity [94].

Reviewing and comparing harvesting techniques reported in the literature comprises a big challenge given the enormous outcome variables and the multiple factors to take into account for each method described (e.g. donor location, type of fat aspiration, pressure, cannula type, etc), therefore not allowing us to give a straightforward answer to the question of which method is the best to assure the highest quality fat graft. Also, when reviewing some of the harvesting methods and pressures applied to collect adipose tissue, we have concluded that most studies focus on endpoints such as in vitro count and viability of cells, however these endpoints have not been proved to translate into better fat graft survival in humans [95].

Optimizing fat grafting methodology in the future is of maximal importance, since patient-related factors are most of the times unchangeable and success may rely almost only on effective fat grafting techniques.

## Ethical approval

N/A.

## Sources of funding

This work was supported by FEDER – Fundo Europeu de Desenvolvimento Regional, through NORTE 2020 Programa Operacional Regional do Norte - NORTE-01-0145-FEDER-000012 and Instituto de Investigação e Inovação em Saúde (Projeto Estratégico UID/BIM/04293/2013).

## Author contribution

Tomás Fontes: data collection and writing the manuscript.

Inês Brandão: data collection, data analysis and writing the manuscript.

Rita Negrão: study design.

Maria João Martins: study design.

Rosário Monteiro: study design and data analysis.

## Conflicts of interest

None to declare.

## Research registration number

N/A.

## Guarantor

Inês Brandão.

Rosário Monteiro.

## Provenance and peer review

Not commissioned, externally peer reviewed.
